# The Effectiveness and Safety of Intensive Lipid-Lowering with Different Rosuvastatin-Based Regimens in Patients at High Cardiovascular Disease Risk: A Nonblind, Randomized, Controlled Trial

**DOI:** 10.31083/j.rcm2408222

**Published:** 2023-08-01

**Authors:** Lili Lin, Sihua Luo, Kuan Cai, Huanliang Huang, Hao Liang, Liqin Zhong, Yunhong Xu

**Affiliations:** ^1^Fever Clinic, The Third Affiliated Hospital of Guangzhou Medical University, 510150 Guangzhou, Guangdong, China; ^2^Department of Cardiology, The Third Affiliated Hospital of Guangzhou Medical University, 510150 Guangzhou, Guangdong, China; ^3^Department of Ultrasound Medicine, The Third Affiliated Hospital of Guangzhou Medical University, 510150 Guangzhou, Guangdong, China; ^4^Department of General Medicine, The Third Affiliated Hospital of Guangzhou Medical University, 510150 Guangzhou, Guangdong, China; ^5^Hunan Provincial Key Laboratory of Traditional Chinese Medicine (TCM) Diagnostics, Hunan University of Chinese Medicine, 410208 Changsha, Hunan, China

**Keywords:** statin, rosuvastatin, PCSK9 inhibitor, ezetimibe, intensive lipid-lowering therapy, cardiovascular disease risk

## Abstract

**Background:**

A statin alone or non-statins as add-ons have been introduced to intensive low-density lipoprotein cholesterol (LDL-C) -lowering therapy in patients at risk for high cardiovascular disease (CVD). The purpose of this study was to evaluate the effectiveness and safety of different rosuvastatin-based regimens for patients at high risk.

**Methods:**

Three hundred patients at high CVD risk were randomly assigned to the statin group (rosuvastatin, 20 mg/d), statin_EZ group (statin 10 mg/d + ezetimibe 10 mg/d), statin_pcsk group (statin 10 mg/d + alirocumab 75 mg/2 weeks) or combine3 group (statin 10 mg/d + ezetimibe 10 mg/d + alirocumab 75 mg/2 weeks). The primary outcome measure was cholesterol levels after 24 weeks of follow-up. Secondary outcomes included safety markers and the proportion of patients achieving the 70 mg/dL (1.8 mmol/L) target for LDL-C. A logistic regression model was performed to explore the factors affecting lipid target achievement.

**Results:**

The total cholesterol (TC) and LDL-C levels in the four groups after treatment were significantly lower than those before treatment. TC and LDL-C levels after treatment were significantly different among the four groups (*p* < 0.05). The levels in both the combine3 and statin_pcsk9 groups were significantly lower than those in the statin and statin_EZ groups (*p* < 0.05), but there was no significant difference between the combine3 and statin_pcsk9 groups. Fifty-one participants (69%) in the statin_pcsk9 group and 56 participants (78%) in the combine3 group achieved the target. Body mass index (BMI) and hypertensive status were related to LDL-C target achievement. The incidence of adverse events in the four groups was low.

**Conclusions:**

The combination of a statin and a PCSK9 inhibitor was safe and more effective for the treatment of high-risk CVD patients, while the addition of ezetimibe was unable to significantly lower lipid levels any further. The rate of achieving the target was higher in patients with hypertension and a low BMI.

**Clinical Trial Registration:**

Chinese Clinical Trial Registry, Identifier: ChiCTR2200058389, Date of Registration: 2022-04-08.

## 1. Introduction

Atherosclerotic cardiovascular disease (ASCVD) is the leading cause of death. Hypercholesterolemia is associated with an increased risk of atherosclerosis (AS) and ASCVD. Clinical studies have shown that high circulating levels of low-density lipoprotein cholesterol (LDL-C) are associated with the development of ASCVD and death and that controlling LDL-C levels reduces the risk of major cardiovascular events [1]. In patients with hypercholesterolemia, lipid-lowering therapy is the cornerstone of primary and secondary prevention of ASCVD [2].

Recently, new concepts and recommendations were presented in the guidelines for lipid-lowering therapy. Based on the prevalence of dyslipidemia complicated with cardiovascular disease (CVD) as well as studies on recommended lipid levels, the 2003–2004 National Health and Nutrition Examination Survey (NHANES) [3] suggested strengthening lipid-lowering combination therapy in individuals with CVD. The American College of Cardiology/American Heart Association (ACC/AHA) 2013 guideline [4] on the treatment of blood cholesterol in adults recommended an LDL-C target of <2.5 mmol/L (97 mg/dL) for patients at high risk of CVD (an elevated risk factor or a score level of 5–10%). The 2016 European Society of Cardiology (ESC) guidelines [5] for the management of dyslipidemias defined familial hypercholesterolemia (FH) patients with high CVD risk requiring intensive lipid-lowering therapy and a stringent LDL-C goal of 2.6 mmol/L (100 mg/dL) or a >50% decrease in LDL-C when target levels cannot be reached. Subsequently, large cardiovascular outcome trials on CVD high-risk patients following guideline-recommended intensive lipid-lowering therapy have proven the benefits of reducing CVD risk and events [6].

Over the past decades, statins, which primarily inhibit hepatic cholesterol synthesis, have been identified as the first-line agents for lipid-lowering therapy to prevent CVD [7]. However, LDL-C-lowering targets for the prevention of CVD have continuously decreased. The competitive inhibition of 3-hydroxy-3-methyl-glutaryl-coenzyme A (HMG-CoA) reductase (HMGR), an enzyme involved in the rate-limiting step in cholesterol biosynthesis in hepatocytes, is the primary molecular mechanism of most statins [8]. Compared with atorvastatin, new statins such as rosuvastatin have produced stronger reductions in LDL-C [9]; achieving such goals in high-risk patients with statins alone is virtually impossible [10]. In addition, patients with high-dose intensive statin monotherapy may experience a high incidence of adverse effects [11], including muscle pain, central nervous system symptoms, liver function abnormalities and diabetic symptoms. In such cases, switching intensive lipid-lowering strategies to other lipid-lowering drugs or combination therapy can be considered [12].

The ACC released a clinical pathway for non-statin therapy for additional reduction of LDL-C in statin-treated patients [13], and cholesterol absorption inhibitors (e.g., ezetimibe), proprotein convertase subtilisin/kexin type 9 (PCSK9) inhibitors, and bile acid sequestrants were chosen as non-statin options for additional reduction of LDL-C. Ezetimibe is a cholesterol absorption inhibitor that prevents the absorption of dietary and biliary cholesterol across the intestinal wall [14, 15, 16]. Alirocumab is monoclonal antibody that blocks the PCSK9 protein from working. As a result, levels of LDL receptors (LDL-R) increase, and lipid levels fall. Moreover, by inhibiting PCSK9, internalized LDL-R can be recycled back to the cell surface, leading to lower LDL-C levels [17]. Statin medications can be combined with PCSK9 inhibitors because they act on different pathways [18]. Several large trials have confirmed the significant beneficial effects of PCSK9 inhibitors on reducing LDL-C levels and CVD risk [19, 20, 21].

A statin plus ezetimibe or a PCSK9 inhibitor was associated with fewer major cardiovascular events than a statin alone [22, 23] and is recommended as intensive lipid-lowering therapy [24]. Which combination is preferable, however, still lacks strong evidence [10]. Herein, we assessed the safety and efficacy of various intensive lipid-lowering regimens based on rosuvastatin, including the addition of ezetimibe or a PCSK9 inhibitor, or both, for treating hypercholesterolemia in high-risk CVD patients. Furthermore, we investigated the factors associated with achieving the lipid-lowering target.

## 2. Materials and Methods

This was a prospective, nonblind, randomized, controlled trial. All patients included in the study, who were at high risk of CVD, were sourced from the Third Affiliated Hospital of Guangzhou Medical University [2022-027]. The protocol was approved by the Institutional Ethics Committee of the hospital, and written informed consent was obtained from all patients before treatment. The trial, registered in the Chinese Clinical Trial Registry (registration number: ChiCTR2200058389), adheres to CONSORT guidelines.

### 2.1 Study Population—Inclusion and Exclusion Criteria

Inpatients and outpatients (at least 18 years of age) at high CVD risk who were treatment naive or receiving non-intensive lipid-lowering therapy were invited to participate in the trial from March 2022 to June 2022. The inclusion criteria were based on the high CVD risk category in 2019 ESC/EAS guidelines for the management of dyslipidemias [25]: patients with markedly elevated single risk factors, in particular TC >8 mmol/L (>310 mg/dL), LDL-C >4.9 mmol/L (>190 mg/dL), or blood pressure (BP) >180/110 mmHg; patients with FH without other major risk factors; patients with diabetes mellitus (DM) without target organ damage, with a DM duration >10 years or another additional risk factor; patients with moderate chronic kidney disease (CKD) (estimated glomerular filtration rate (eGFR) 30–59 mL/min/1.73 $m^{2}$); and patients with an ESC calculated SCORE >5% and <10% for a 10-year risk of fatal CVD. All patients underwent carotid ultrasound, and the presence of carotid atherosclerosis was determined based on the presence of an atheromatous plaque at carotid crossings and an increase in carotid intima-media thickness (IMT) [26].

Exclusion criteria were as follows: (1) secondary hyperlipidemia due to thyroid abnormalities, nephrotic syndrome or drug use; (2) the presence of severe cardiac insufficiency, defined as a left ventricular ejection fraction (EF) ≤40%; (3) pregnancy; (4) abnormal liver function, defined as glutamic aminotransferase (AST) and glutamic aminotransferase (ALT) levels three or more times above the upper reference limit of normal; (5) the presence of connective tissue disease, rheumatic/immune system diseases, rhabdomyolysis, hematological disorders or malignancies, or intracranial lesions; (6) a history of major surgery, trauma, acute or chronic infection or fever within the past month; and (7) the presence of clear contraindications or a history of allergy to either drug.

### 2.2 Study Design

**Fig. 1.**
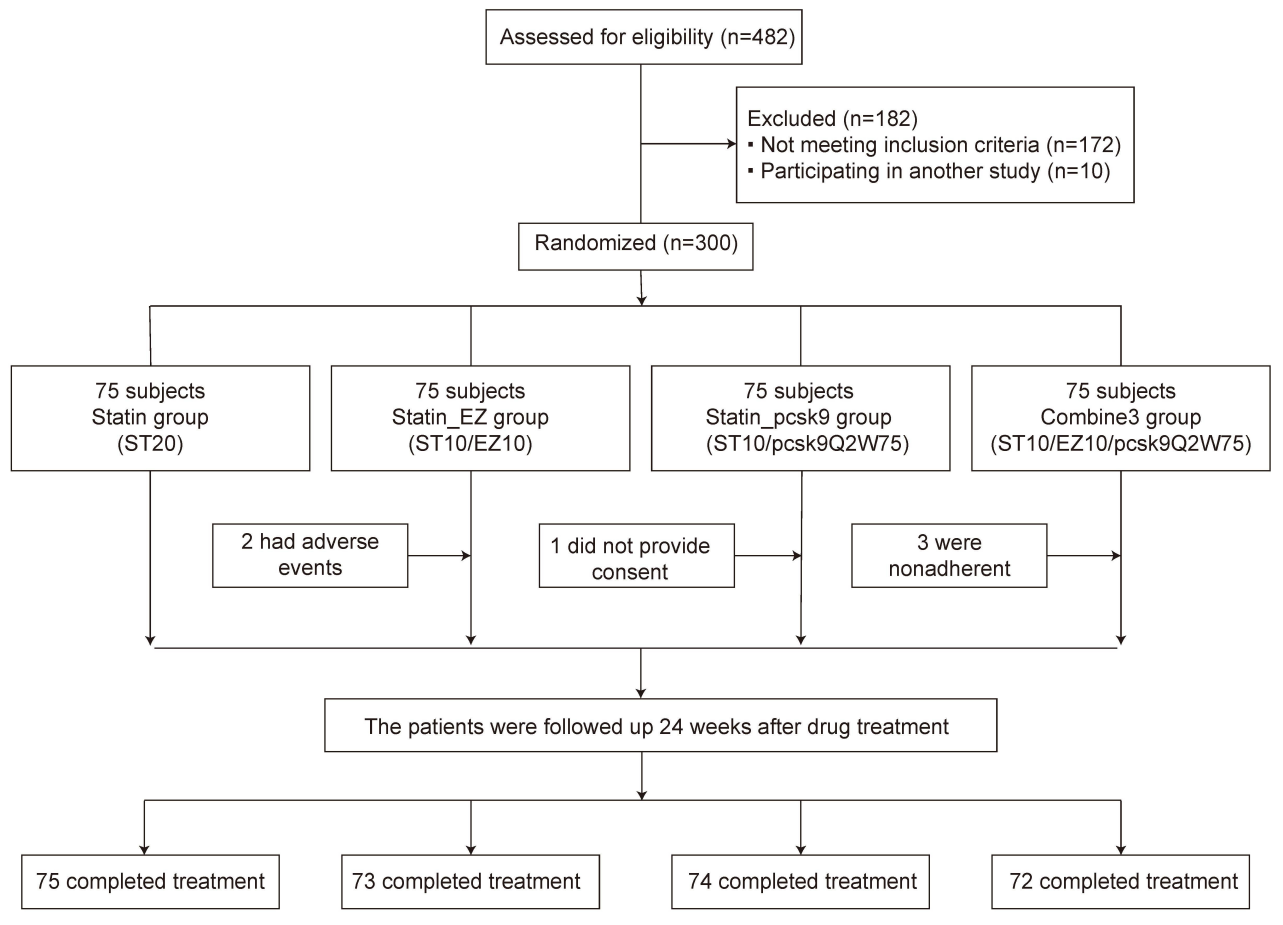
**Flow chart of patient enrollment in the study**. Statin_EZ group: statin + ezetimibe; statin_pcsk9 group: statin + a PCSK9 inhibitor; combine3: statin + ezetimibe + a PCSK9 inhibitor; ST20: statin at 20 mg daily; ST10/EZ10: statin at 10 mg plus ezetimibe at 10 mg daily; ST10/pcsk9Q2W75: statin at 10 mg daily plus a pcsk9 inhibitor at 75 mg every 2 weeks; ST10/EZ10/pcsk9Q2W75: statin at 10 mg plus ezetimibe at 10 mg daily and a PCSK9 inhibitor at 75 mg every 2 weeks.

Statistical assistants at our medical center used R software (ver. 4.2.1, The R Foundation, Vienna, Austria) to generate the assignment list and sealed envelopes to store the random numbers. Participants meeting the eligibility criteria were screened and enrolled. The randomization and allocation procedures were carried out by the assistant based on the random number obtained from the sealed envelopes. All eligible patients were consistently provided with standard treatments for their underlying conditions, as well as advice on dietary modifications and control, including abstaining from smoking and alcohol, following a low-fat diet, and engaging in appropriate exercise. All selected patients were assigned (1:1:1:1) to the rosuvastatin 20 mg/d (statin) group, rosuvastatin 10 mg/d + ezetimibe 10 mg/d (statin_EZ) group, rosuvastatin 10 mg/d + alirocumab 75 mg/2 weeks (statin_pcsk9) group and rosuvastatin 10 mg/d + ezetimibe 10 mg/d + alirocumab 75 mg/2 weeks (combine3) group (Fig. 1).

Laboratory tests, including tests for lipid levels (LDL-C, total cholesterol (TC), triglycerides (TGs), and high-density lipoprotein cholesterol (HDL-C)), liver and renal function tests, and creatine kinase (CK) tests, were conducted prior to the treatment and at the 12th and 24th weeks. Information on sex, age, smoking history, hypertension, diabetes history, body mass index (BMI) and other related medical history was collected. The patients were followed up for 24 weeks.

### 2.3 Outcome Measures

Changes in lipid levels from baseline at 24-week follow-up were the primary outcome measure. Secondary outcome measures were the percentage of patients at the 24th week who achieved the LDL-C target of <70 mg/dL (<1.8 mmol/L) recommended in the 2019 ESC/EAS guidelines for the management of dyslipidemias [25]. Cardiovascular events (including death, myocardial infarction, stroke, heart failure, etc.) were also recorded.

### 2.4 Safety

Safety was assessed in the study through reports of treatment-induced adverse events (TEAEs) (defined as any adverse reaction during the period [27], including drug allergies, local injection site reactions, cardiovascular or diabetic complication events, etc.) and vital sign and laboratory parameter abnormalities.

### 2.5 Statistical Analysis

R software (ver. 4.2.1, The R Foundation, Vienna, Austria) was used for statistical analysis. Normally distributed data are presented as the mean ± standard deviation (SD), and *t* tests were performed for comparisons between two groups. Non-normally distributed data are presented as the median [range], and Kruskal‒Wallis one-way ANOVA was performed for comparisons between multiple groups, whereas a nonparametric test (Dunn’s test) with *p* value adjustment (holm) was performed for multiple comparisons. Categorical data are presented as frequencies or percentages, and comparisons between groups were made using the $\chi^{2}$ test. The change in each lipid index from baseline to 24 weeks of treatment was evaluated using a nonparametric test (Wilcoxon signed-rank test) and the rank-biserial correlation was applied for non-parametric tests of differences. A logistic regression model was constructed for exploring factors related to achieving the LDL-C target. Adjustment variables were selected from 2019 ESC/EAS guidelines [25] for the rationales of nonlipid targets, including smoking history, hypertension, diabetes history and BMI. Odds ratios (ORs) and 95% confidence intervals (CIs) were calculated. *p* < 0.05 was considered statistically significant.

The sample size calculation was performed using R software (pwr package, ver. 1.3). The sample size used in the present study was satisfactory based on the result of calculation (α = 0.05, β = 0.05). Details can be found in **Supplementary File 1**.

## 3. Results

### 3.1 Clinical Characteristics

Initially, 300 patients participated in the lipid-lowering therapy session. However, two patients in the statin_EZ group were withdrawn from therapy due to adverse drug reactions (nausea and rashes). Additionally, one patient in the statin_pcsk9 group was withdrawn due to insufficient data, and three patients in the combine3 group were withdrawn due to non-adherence to the treatment. Therefore, 294 patients were included for analyses. At baseline, the sex distribution and patient characteristics such as treatment naive, age, smoking, hypertension, diabetes, BMI, and lipid levels were generally similar among the groups (*p* > 0.05) (Table 1).

**Table 1.** **Baseline characteristics of the study population**.

Variable		N	combine3	statin	statin_EZ	statin_pcsk9	*p* value
			N = 72	N = 75	N = 73	N = 74	
Sex		294					0.20
	Female	141	27 (38%)	40 (53%)	39 (53%)	35 (47%)	
	Male	153	45 (62%)	35 (47%)	34 (47%)	39 (53%)	
Treatment naive		88	23 (32%)	26 (35%)	21 (28%)	18 (24%)	0.55
Age			68 [37–88]	70 [50–93]	67 [45–97]	71 [48–87]	0.13
HBP		244	57 (79%)	65 (87%)	61 (84%)	61 (82%)	0.70
Smoking		98	27 (38%)	25 (33%)	23 (32%)	23 (31%)	0.80
DM		120	30 (42%)	30 (40%)	29 (40%)	31 (42%)	0.94
BMI			23.6 [17.3–34.6]	24.0 [18.6–33.6]	23.5 [16.2–36.7]	24.0 [18.8–33.3]	0.70
TC			4.20 [1.51–7.18]	3.96 [2.36–6.89]	4.20 [1.85–7.26]	3.92 [2.41–6.41]	0.13
TGs			1.39 [0.39–4.31]	1.30 [0.38–5.54]	1.42 [0.59–6.40]	1.46 [0.55–4.99]	0.80
LDL-C			2.71 [0.26–4.86]	2.52 [1.05–4.31]	2.76 [0.64–5.60]	2.37 [0.71–4.86]	0.20
HDL-C			1.16 [0.65–2.26]	1.22 [0.61–2.17]	1.11 [0.72–2.40]	1.08 [0.14–1.87]	0.30

Abbreviations: HBP, high blood pressure; DM, diabetes mellitus; BMI, body mass index; TC, total cholesterol; TGs, triglycerides; LDL-C, low-density lipoprotein cholesterol; HDL-C, high-density lipoprotein cholesterol.

### 3.2 Pretreatment vs. Posttreatment Effects

**Fig. 2.**
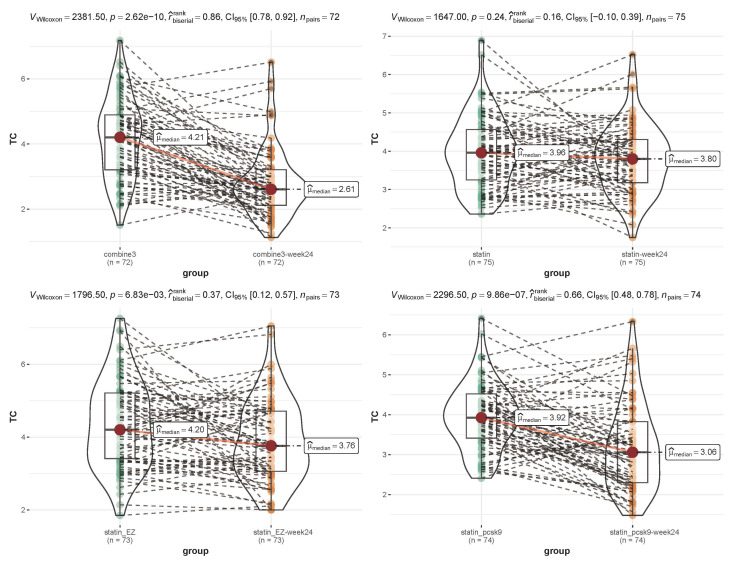
**Comparison of the total cholesterol level before and after treatment within each group**. Combine3 group: statin (10 mg/d) + ezetimibe (10 mg/d) + the PCSK9 inhibitor alirocumab (75 mg Q2W); statin_pcsk9 group: statin (10 mg/d) + the PCSK9 inhibitor alirocumab (75 mg Q2W); statin group: rosuvastatin (20 mg); statin_EZ group: statin (10 mg/d) + ezetimibe (10 mg/d). A nonparametric test (Wilcoxon signed-rank test) and the rank-biserial correlation was applied for non-parametric tests of differences. TC, total cholesterol.

**Fig. 3.**
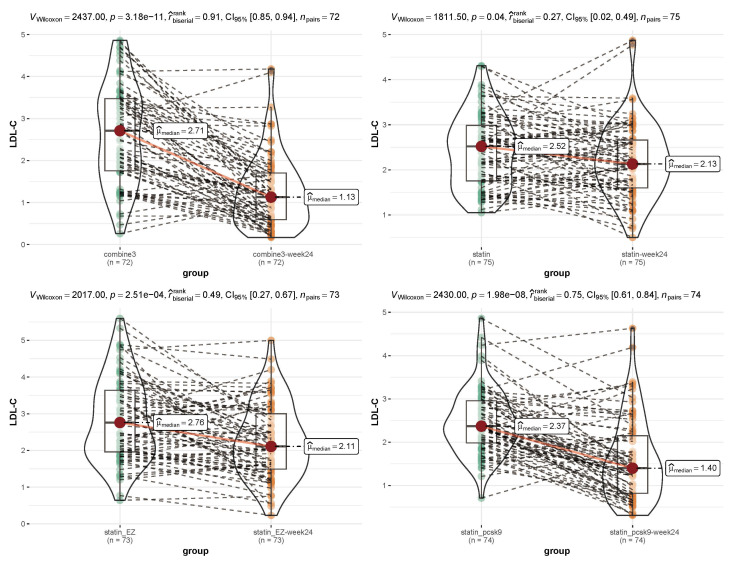
**Comparison of the low-density lipoprotein cholesterol (LDL-C) level before and after treatment within each group**. Combine3 group: statin (10 mg/d) + ezetimibe (10 mg/d) + the PCSK9 inhibitor alirocumab (75 mg Q2W); statin_pcsk9 group: statin (10 mg/d) + the PCSK9 inhibitor alirocumab (75 mg Q2W); statin group: rosuvastatin (20 mg); statin_EZ group: statin (10 mg/d) + ezetimibe (10 mg/d). A nonparametric test (Wilcoxon signed-rank test) and the rank-biserial correlation was applied for non-parametric tests of differences.

As shown in Figs. 2,3, before and after treatment, the median level of TC in the combine3 group changed from 4.21 to 2.61 mmol/L (*p* < 0.05), and the median level of LDL-C changed from 2.71 to 1.13 mmol/L (*p* < 0.05). The median level of TC in the statin-pcsk9 group changed from 3.92 to 3.06 mmol/L (*p* < 0.05), and the median level of LDL-C changed from 2.37 to 1.40 mmol/L (*p* < 0.05). Overall, TC and LDL-C levels were significantly lower in both the combine3 and statin-pcsk9 groups after treatment than before treatment. In addition, the decrease in lipid levels from pretreatment to posttreatment was sharper in the combine3 group than in the statin-pcsk9 group (TC: rank-biserial = 0.86 vs. 0.66, LDL-C: rank-biserial = 0.91 vs. 0.75). Moreover, the HDL-C levels were significantly higher than pretreatment in combine3 and statin-pcsk9 groups (**Supplementary File 2**).

### 3.3 Lipid-Lowering Effect between Groups

**Fig. 4.**
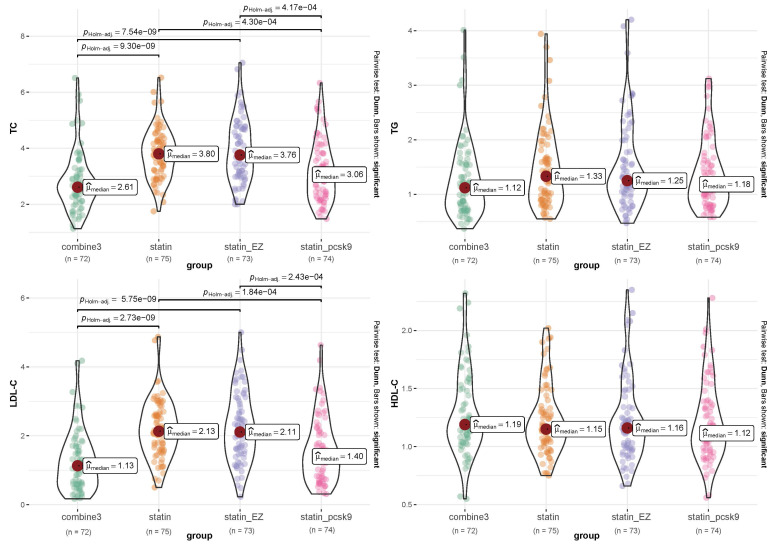
**Differences in lipid levels among groups after the 24-week treatment**. Combine3 group: statin (10 mg/d) + ezetimibe (10 mg/d) + the PCSK9 inhibitor alirocumab (75 mg Q2W); statin group: rosuvastatin (20 mg); statin_EZ group: statin (10 mg/d) + ezetimibe (10 mg/d); statin_pcsk9 group: statin (10 mg/d) + the PCSK9 inhibitor alirocumab (75 mg Q2W). *p* values are for pairwise comparisons between the combine3 and comparator groups within each baseline regimen. There were significant differences among the four groups in TC and LDL (*p* value < 0.05), and *p* values are shown for descriptive purposes only. TC, total cholesterol; LDL-C, low-density lipoprotein cholesterol; HDL-C, high-density lipoprotein cholesterol.

As shown in Fig. 4, the TC levels after 24 weeks of treatment were 2.61 [1.13–6.51] mmol/L in the combine3 group, 3.80 [1.75–6.52] mmol/L in the statin group, 3.76 [2.00–7.05] mmol/L in the statin_EZ group, and 3.06 [1.48–6.33] mmol/L in the statin_pcsk9 group. There was a significant difference among the four groups (*p* < 0.05). The levels in both the combine3 and statin_pcsk9 groups were significantly lower than those in the statin and statin_EZ groups (*p* < 0.05), but there was no significant difference between the combine3 and statin_pcsk9 groups, nor between the statin and statin_EZ groups.

Similarly, there was significant difference among the four groups in LDL-C (*p* < 0.05). The level of LDL-C in the combine3 group was significantly lower than that in either the statin or statin_EZ groups (*p* < 0.05), while no significant difference was found between the combine3 and statin_pcsk9 groups. There were no significant differences among the four groups in TGs or HDL-C.

### 3.4 Factors related to Achieving the Target

**Fig. 5.**
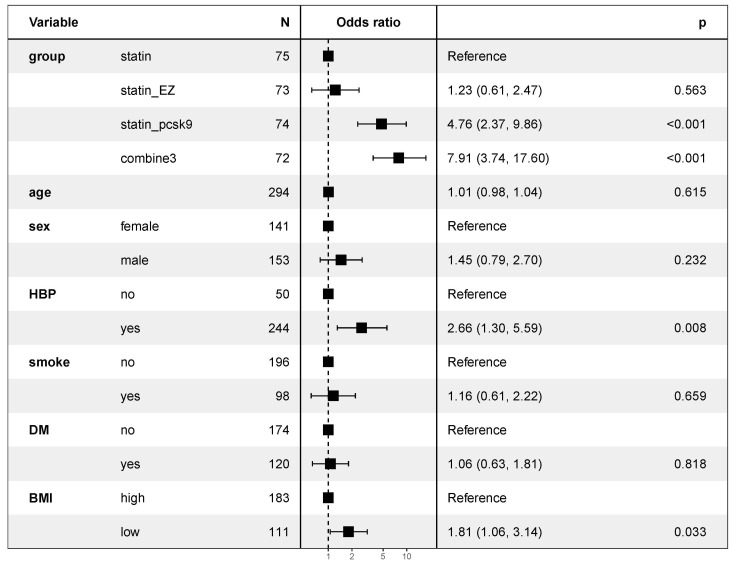
**Multivariate logistic regression analysis for factors related to achieving the target**. EZ, ezetimibe; PCSK9, PCSK9 inhibitor alirocumab; combine3, statin + ezetimibe + alirocumab; HBP, high blood pressure; DM, diabetes mellitus; BMI, body mass index.

Overall, 160 patients achieved the target (<70 mg/dL (1.8 mmol/L)) after treatment. Fifty-one participants (69%) in the statin_pcsk9 group and 56 participants (78%) in the combine3 group achieved the target (Table 2). The results of the multivariate logistic regression analysis for treatment group, age, sex, hypertension, smoking, diabetes and BMI (BMI ≥23 ${\mathrm{kg/m}}^{2}$ was categorized as high BMI) are shown in Fig. 5. The combine3 group (OR 7.91, 95% CI: 3.74–17.60, *p* < 0.05) and the statin-pcsk9 group (OR 4.76, 95% CI: 2.37–9.86, *p* < 0.05) were associated with achieving the target. Hypertensive status was associated with achieving the target, and patients with hypertension were inclined to achieve the goal (OR 2.66, 95% CI: 1.30–5.59, *p* = 0.008). BMI was also related to achieving the target, and the patients with a low BMI (OR 1.81, 95% CI 1.06–3.14, *p* = 0.033) more frequently reached the target. Hypertensive status and BMI were independent predictive factors for achieving the target, while other factors were not related to achieving the target.

**Table 2.** **The proportion of target achievement in different groups**.

Variable	N	combine3	statin	statin_EZ	statin_pcsk9	*p* value
		N = 72	N = 75	N = 73	N = 74	
Goal achieved	160	56 (78%)	25 (33%)	28 (38%)	51 (69%)	<0.001

### 3.5 Safety

A total of six patients experienced adverse events during the 24-week follow-up period. Three of the patients in the statin group experienced mild muscle pain, a common side effect of statins. Two patients in the statin_pcsk9 group had a rash at the injection site, and one patient also had a mild rash at the injection site in the combined 3 group caused by injection-site reactions, but all such side effects were mild and did not lead to a discontinuation of treatment. There were no groups with AST, ALT, or CK levels three or more times the upper limit of normal during the follow-up period. Overall, the incidence of adverse events that may have been related to the study drug was low in all four groups. During the study period, patients in all groups tolerated the drug well, with no serious adverse events, permanent treatment discontinuations, deaths, myocardial infarction or stroke. Additionally, there were no cases of hemolytic anemia or diabetic complications.

## 4. Discussion

This was a nonblind, randomized, controlled trial to evaluate the effectiveness and safety of different intensive lipid-lowering therapy regimens based on statins for high-risk CVD patients. The results showed that the combination of a statin and a PCSK9 inhibitor was more effective than other statin-based regimens for the treatment of high-risk CVD patients in terms of lowering the lipid levels and achieving the target. Although the lipid levels in the combine3 group were lower than those in the statin_pcsk9 group, there was no significant difference between the two groups. The combination of a statin and a PCSK9 inhibitor was sufficient, and the addition of ezetimibe was unable to significantly lower lipid levels any further. Multivariate analysis demonstrated that BMI and hypertensive status were related to the lipid-lowering effect. The rate of achieving the target was higher in patients with vs. without hypertension, and patients with a low BMI were likely to reach the goal. While only eight patients experienced adverse events, two of whom were withdrawn from the trial on account of drug-induced nausea and rashes, most patients tolerated the drugs well during the study period.

Based on the findings of several key trials, guidelines increasingly recommend intensive lipid-lowering therapy, and the target levels decrease with the stratification of ASCVD risk. Statins, as first-line drugs for lipid-lowering therapy in CVD, often fail to ensure that patients achieve their individual LDL-C target levels. In particular, patients with ASCVD have poor outcomes when basic statin therapy fails to achieve LDL-C treatment goals [28, 29, 30]. Although IMPROVE-IT trial [31] and a Chinese study showed that the combination of a statin and ezetimibe was superior to a statin alone, with a greater effect on lowering LDL-C in patients after acute coronary syndrome (ACS) [7], a statin plus ezetimibe in our trial did not show superior effects in lipid reduction. This discrepancy may be attributable to the differences in the enrolled populations: Most ACS patients are aware of life-threatening status and therefore have a strong incentive to modify their lifestyle and to adhere more closely to treatment regimens. Conversely, lipid reduction in CVD high risk patients is more challenging, as FH, DM and moderate CKD invariably cause dyslipidemia, and patients are typically less motivated to modify lifestyle.

Several studies demonstrated that the LDL-C-lowering effect of the PCSK9 inhibitor alirocumab added to the effect of maximally tolerated statins (± other lipid-lowering therapy) and decreased the cardiovascular risk of patients [21, 27, 32, 33, 34, 35]. The result of the ODYSSEY ALTERNATIVE randomized trial [36] showed a 45% reduction in LDL in the alirocumab group as opposed to a 15% reduction in LDL in the ezetimibe group. In the ODYSSEY OPTIONS trial [37], LDL-C was reduced by 16.3% at 24 weeks with 20 mg of rosuvastatin, by 14.4% with ezetimibe and by 50.6% with alirocumab. Similarly, in this trial, LDL-C levels were reduced by 58.3% and 40.9% in the statin_pcsk9 and combine3 groups, respectively. Significant increase of HDL-C level in PCSK9 inhibitor-related treatment groups indicate the dual-efficacy of decreasing LDL-C and increasing HDL-C. HDL-C is beneficial to health that promotes rapid atherosclerosis regression [38]. This may explain why PCSK9 inhibitor rapidly reduces plaque lipid content [39]. The correlation between the HDL-C elevation effect from PCSK9 inhibitor and plaque morphology by imaging should be further investigated.

Furthermore, it is important to consider the effects of different lipid-lowering therapies as well as the safety of lipid regulation. Adverse reactions to lipid-lowering drugs generally include muscle pain, drug hypersensitivity, cardiovascular pathology, central nervous system symptoms, abnormal liver function, and diabetic symptoms. The findings of a meta-analysis suggest that an increased risk of hemorrhagic stroke is associated with more intensive LDL-C-lowering statin treatments, which may be exacerbated by high-intensity statin use [40]. In our study, the total proportion of adverse drug events was approximately 2%, with no significant differences in AST, ALT, CK, or Scr in any group. In its evaluation of safety and efficacy, the Odyssey Mono study found that alirocumab significantly reduced the rate of major adverse cardiovascular events compared to the control group (1.7% vs. 3.3%). In the ODYSSEY OPTIONS [27] randomized trial, a lower rate of major adverse cardiovascular events was more fully achieved with the addition of alirocumab to a statin therapy than with other lipid-lowering treatments alone. The results from the ODYSSEY OUTCOMES randomized controlled trial [41] showed that alirocumab was well tolerated in all subgroups, defined by the presence of metabolic risk factors; adding alirocumab to a statin in combination with ezetimibe not only increased the lipid-lowering effect but also significantly reduced the risk of major adverse cardiovascular events (MACEs). The findings of these studies demonstrate the safety advantages of the addition of alirocumab in the context of statin therapy compared to other types of lipid-lowering therapy.

According to our research, 51 participants (69%) in the statin_pcsk9 group and 56 participants (78%) in the combine3 group achieved the target, in line with previous studies [32, 34]. Even with high-intensity treatment, a third of individuals were above the target, perhaps due to unknown FH status [42] and possibility of poor adherence [43]. In our multivariate analysis, the influence of hypertensive status on lipid-lowering effects showed that patients with hypertension were more likely to achieve the stated goal. This may be related to the side effects of antihypertensive drugs on lipid metabolism. For example, diuretic drugs such as hydrochlorothiazide can weaken the inhibitory effect of insulin on lipolysis, strengthen lipolysis, increase free fatty acids in the blood, and lead to abnormal blood lipid levels [44]. However, the incorporation of an angiotensin-converting enzyme (ACE)-inhibitor or a calcium channel blocker and a statin has consistently demonstrated reductions in lipid levels and the number of ASCVD events in patients with hypertension and lipid disorders [45, 46]. Unfortunately, since we did not collect sufficient data regarding antihypertensive drug use for the enrolled patients with hypertension, the mechanism is merely hypothetical. Logistic analysis showed that the rate of achieving the goal was higher in patients with a low BMI, as anticipated, since low body weight is associated with a low-fat burden and consequently a reduction in lipid levels [47].

Finally, several limitations of this study should be noted. As a single-center study, the sample size was relatively small. Because the patients were treated with different drugs and administration routes (e.g., statins are oral medication, but PCSK9 inhibitors are given by local injection), the blinding method is challenging to perform, which may result in biases during the trial. In addition, the evaluated factors influencing the achievement of lipid targets were limited and require further investigation.

## 5. Conclusions

In summary, in the treatment of high-risk CVD patients, the combination of a statin and a PCSK9 inhibitor was safe and more effective in lowering lipid levels and achieving the target than other rosuvastatin-based regimens, while the addition of ezetimibe was unable to significantly lower lipid levels any further. The rate of achieving the target was higher in patients with hypertension and low BMI.

## Data Availability

The authors declare that the data supporting the findings of this study are available within the article and its supplementary files. The original data can be download from https://doi.org/10.5061/dryad.0rxwdbs4b.

## Supplementary Material

Supplementary material associated with this article can be found, in the online version, at https://doi.org/10.31083/j.rcm2408222.
