# Supplementary material for: The Effectiveness and Safety of Intensive Lipid-Lowering with Different Rosuvastatin-Based Regimens in Patients at High Cardiovascular Disease Risk: A Nonblind, Randomized, Controlled Trial
Source: Rev Cardiovasc Med. 2023 Aug 1;24(8):222. doi: 10.31083/j.rcm2408222 (PMC11262440; doi:10.31083/j.rcm2408222)
Supplement: Supplementary file 1 [file 2153-8174-24-8-222-s1.zip › Supplementary File 1.pdf]

Sample size Calculation

The Sample Size Calculator (https://www.statskingdom.com/sample_size_all.html) for ANOVA was used. The calculator is created based on the R package(pwr). The detailed parameters are in the following figure.

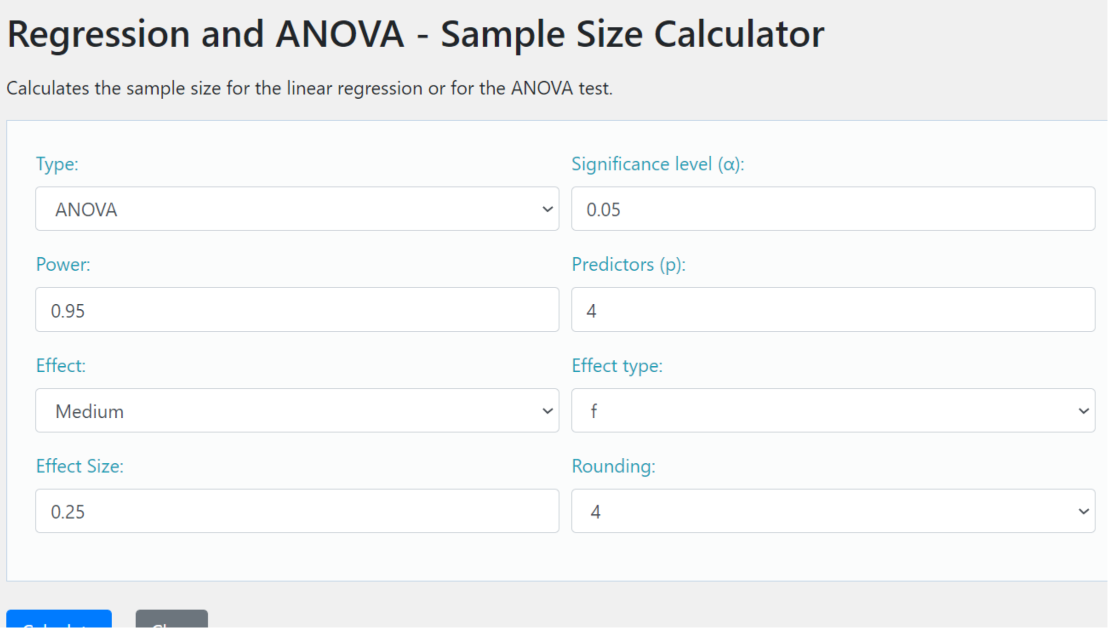

Results

A sample size of 279 (at least 69 in each group) results in a statistical test power of 0.9503.

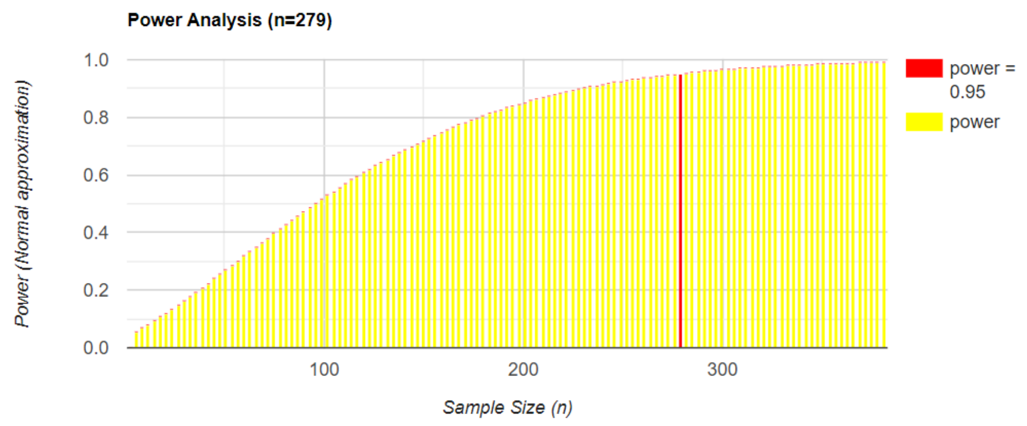

n=2 power=0
n=4 power=0
n=8 power=0.06369
n=16 power=0.09719
n=32 power=0.172
n=64 power=0.3376
n=128 power=0.6388
n=256 power=0.9302
n=512 power=0.999
n=278 power=0.9495

R code

```
rm(list = ls())
if(!"pwr" %in% installed.packages()){install.packages("pwr")}
library(pwr)
results=pwr.anova.test(k =4, power=0.95, f=0.25, sig.level =0.05)
results
total_n = ceiling(results$n*4)
total_n
```
