# Supplementary material for: The Effectiveness and Safety of Intensive Lipid-Lowering with Different Rosuvastatin-Based Regimens in Patients at High Cardiovascular Disease Risk: A Nonblind, Randomized, Controlled Trial
Source: Rev Cardiovasc Med. 2023 Aug 1;24(8):222. doi: 10.31083/j.rcm2408222 (PMC11262440; doi:10.31083/j.rcm2408222)
Supplement: Supplementary file 1 [file 2153-8174-24-8-222-s1.zip › Supplementary File 2.pdf]

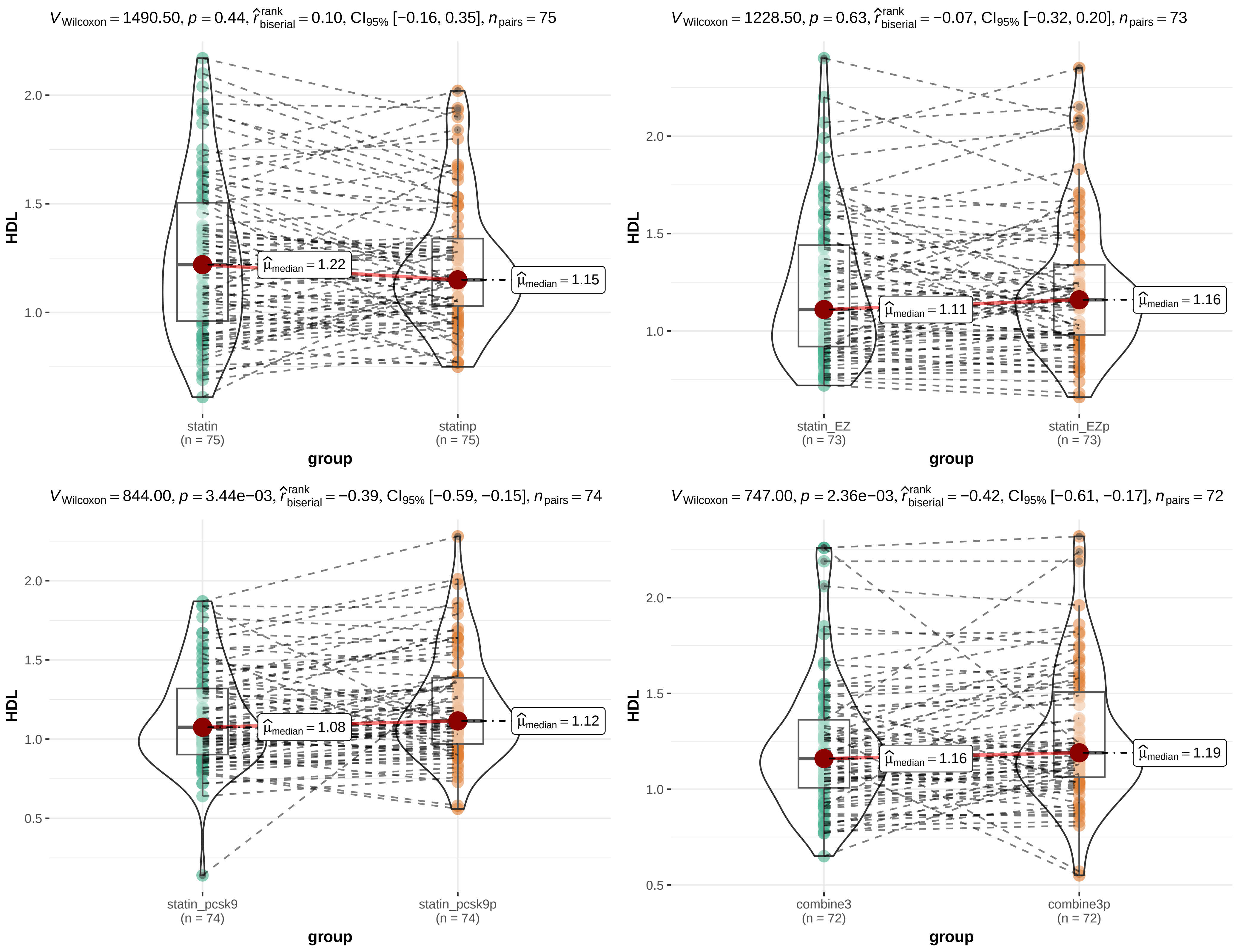

V Wilcoxon = 1490.50, p = 0.44, r̂ rank biserial = 0.10, CI95% [−0.16, 0.35], n pairs = 75
HDL
2.0
1.5
1.0
μ̂ median = 1.22
μ̂ median = 1.15
statin
(n = 75)
statinp
(n = 75)
group
V Wilcoxon = 1228.50, p = 0.63, r̂ rank biserial = −0.07, CI95% [−0.32, 0.20], n pairs = 73
HDL
2.0
1.5
1.0
μ̂ median = 1.11
μ̂ median = 1.16
statin_EZ
(n = 73)
statin_EZp
(n = 73)
group
V Wilcoxon = 844.00, p = 3.44e−03, r̂ rank biserial = −0.39, CI95% [−0.59, −0.15], n pairs = 74
HDL
2.0
1.5
1.0
0.5
μ̂ median = 1.08
μ̂ median = 1.12
statin_pcsk9
(n = 74)
statin_pcsk9p
(n = 74)
group
V Wilcoxon = 747.00, p = 2.36e−03, r̂ rank biserial = −0.42, CI95% [−0.61, −0.17], n pairs = 72
HDL
2.0
1.5
1.0
0.5
μ̂ median = 1.16
μ̂ median = 1.19
combine3
(n = 72)
combine3p
(n = 72)
group
